# Experimental and Molecular Dynamics Study of Pyrite Effects on the Flocculation of Clayey Tailings in Seawater

**DOI:** 10.3390/polym17212895

**Published:** 2025-10-29

**Authors:** Steven Nieto, Eder Piceros, Gonzalo R. Quezada, Fernando Betancourt, Pedro Robles, Williams Leiva, Ricardo I. Jeldres

**Affiliations:** 1 Water Research Center for Agriculture and Mining (CRHIAM), Universidad de Concepción, Victoria 1295, Concepción 4030000, Chile; yeison.nieto.mejia@ua.cl; 2 Faculty of Engineering and Architecture, Universidad Arturo Prat, Iquique 1100000, Chile; edpicero@unap.cl; 3 Departamento de Ingeniería de Procesos y Bioproductos, Facultad de Ingeniería, Universidad del Bío-Bío, Concepción 4030000, Chile; grquezada@ubiobio.cl; 4 Departamento de Ingeniería Metalúrgica, Universidad de Concepción, Concepción 4030000, Chile; fbetancourt@udec.cl; 5 Escuela de Ingeniería Química, Pontificia Universidad Católica de Valparaíso, Valparaíso 2340000, Chile; pedro.robles@pucv.cl; 6 Facultad de Ingeniería, Universidad San Sebastián, Sede Concepción, Concepción 4030000, Chile; williams.leiva@uss.cl; 7 Departamento de Ingeniería Química y Procesos de Minerales, Facultad de Ingeniería, Universidad de Antofagasta, Antofagasta 1240000, Chile; 8 Advanced Mining Technology Center (AMTC), Universidad de Antofagasta, Antofagasta 1240000, Chile

**Keywords:** pyrite flocculation, anionic polyacrylamide, clay-based tailings, seawater thickening, molecular dynamics simulations, tailings management

## Abstract

This study investigates the effect of pyrite content on the flocculation and sedimentation of clay-based tailings composed of kaolin, quartz, and pyrite in seawater at pH 8. A high-molecular-weight anionic hydrolyzed polyacrylamide (SNF 704) was used in batch settling tests, supported by floc characterization with FBRM, zeta potential measurements, and molecular dynamics (MD) simulations. Results showed that increasing pyrite content reduced the maximum floc size and increased the fraction of unflocculated fines, particularly at 10 g/t dosage. Although the fractal dimension remained nearly constant (1.92–1.97 at 10 g/t and 2.05–2.08 at 30 g/t), floc density increased linearly with pyrite proportion due to its higher specific gravity. Zeta potential analysis confirmed strong polymer–pyrite interactions, with charge inversion from +5.3 to −4.5 mV, while MD simulations indicated that adsorption occurs mainly through aliphatic chain segments, in contrast to hydrogen bonding observed for quartz and kaolinite. These findings demonstrate that pyrite affects flocculation dynamics both by its density and by specific polymer–surface interactions, directly influencing floc size, density, and sedimentation performance in seawater thickening systems.

## 1. Introduction

The limited availability of freshwater in arid and semi-arid regions has driven the increasing use of seawater as an alternative resource in various stages of mineral processing [1]. While this measure responds to an environmental need, it also entails significant operational challenges, since the ionic composition of seawater modifies the physicochemical properties of flocculation systems, altering the interaction between mineral particles and flocculants, influencing the efficiency of the thickening operation [2]. Thickening aims to increase the concentration of solids in a pulp through gravitational sedimentation. This process is accelerated by the addition of flocculant chemical reagents, which favor the formation of larger and heavier flocs [3]. This allows an efficient separation of clear water from solid material, generating a denser sludge in the thickener underflow that is transported by pipelines and deposited in tailings storage dams, and an overflow of clarified water that is recovered and recycled in upstream operations.

Among the flocculants used in copper tailings thickening operations, hydrolyzed anionic polyacrylamide (HPAM) has proven to be one of the most effective. Its superior performance compared to cationic and non-ionic flocculants is attributed to its high capacity to form interparticle bridges, which favors the generation of stable flocs. This characteristic significantly improves the sedimentation rate, optimizes the recovery of clarified water, and facilitates the hydraulic handling of tailings to tailings storage facilities (TSFs) [4,5]. However, its effectiveness in seawater can be significantly influenced by the presence of cations [6], which can promote flocculation by generating additional bonds between the anionic groups of the polyelectrolyte and the surfaces of quartz and kaolin minerals. The formed cation bridges can increase the adsorption density of the reagent, thus improving flocculation efficiency [7]. However, high-salinity conditions can reduce flocculant effectiveness by limiting the radius of gyration of polyelectrolytes, since cations shield the charge of anionic functional groups. This protection prevents molecules from maintaining their extended shape in solution, limiting their ability to form efficient polymer bridges [8]. Nieto et al. [9] evaluated the flocculation of synthetic tailings composed of quartz and kaolinite using an anionic polyacrylamide in seawater and industrial water. For kaolinite, it was observed that high salinity in seawater reduces flocculant effectiveness due to polymer chain coiling. However, the presence of cations favored the formation of more compact flocs through cation bridges, improving the capture of fine particles. This was reflected in denser structures, although with lower sedimentation velocities compared to the system in industrial water (0.05 M CaCl_2_ and 0.01 M NaCl).

Liu et al. [10] evaluated kaolinite sedimentation in low-, medium-, and high-salinity waters using an anionic flocculant Magnafloc 1011 (BASF, Brandford, UK). They observed that, with increasing salinity, the sedimentation rate decreased, despite the presence of the polymer. This effect was attributed to the formation of open edge-face flocs that retain water, in contrast to the compact face-face structures observed in low salinity water, where flocculation and sedimentation were more efficient. Ji et al. [11] studied the effect of salinity on kaolinite flocculation and sedimentation, using 3 types of flocculants: neutral polyacrylamide (PAM), an inorganic-organic hybrid flocculant (Al(OH)3-PAM), and Magnafloc 1011, an anionic acrylamide-acrylate copolymer. The tests were carried out in saline water (Na^+^: 5.832; Mg^2+^: 701; K^+^: 275; Ca^2+^: 679 ppm) and fresh water (Na^+^: 141; Mg^2+^: 7.87; K^+^: 12.5; Ca^2+^: 11.4 ppm), evidencing significant differences in the ionic strength of both media. It was observed that the Magnafloc 1011 flocculant, owing to its higher anionic charge density and high molecular weight, promoted more effective adsorption onto kaolinite surfaces and favored the formation of larger and more settleable flocs.

The effectiveness of the flocculant also depends on the relative proportion and surface characteristics of the minerals present in the tailings. In copper mining in Chile, the mineralogical composition consists of a complex mixture of residual minerals from the concentration process, including clays, quartz, and pyrite. The presence of these minerals in the tailings has a direct impact on the efficiency of the flocculants used in thickening processes such as HPAM, and therefore, on water recovery. The literature addresses research related to the effect on flocculation of clays from the kaolin group [12,13,14]. These clays, mainly composed of kaolinite and a density close to 2600 Kg/m^3^, are non-expansive minerals with a laminar structure, a high area-to-surface ratio, and charge densities that vary on their edges and faces [15]. In the presence of seawater, calcium and magnesium ions compress the electrical double layer of kaolin particles, promoting aggregation and improving process efficiency [16]. However, its high specific surface area may require higher flocculant doses under high concentration conditions, especially when combined with other minerals. Quartz, on the other hand, has a relatively inert surface, which responds well to flocculation, but does not contribute significantly to the structure of the flocs. Iron sulfide or pyrite, with its high density (≈5000 Kg/m^3^), exhibits a surface with reactive sites and different adsorption behavior compared to the kaolin surface. This can influence the formation of flocculent bridges and modify the morphology of the flocs. For example, it may present porous flocs in the presence of flocculant, which reduces the flocculation efficiency in sedimentation in the presence of water with low salt concentration (0.01 M NaCl and 0.005 M CaCl_2_) [17].

Although there are studies analyzing the interaction between flocculants and individual minerals, such as clays or sulfides, the combined influence of kaolin and pyrite on flocculant efficiency in seawater at pH 8 has not been systematically addressed. Recently, Nieto et al. [17] evaluated the relationship between floc structure and flocculation performance of an anionic polyacrylamide SNF 704 (SNF Floerger, Andrézieux, France) in synthetic tailings containing pyrite, using industrial water at pH 10.5. It was observed that pyrite has a high affinity for the flocculant, which generates train-type adsorption that limits the formation of polymeric bridges, resulting in more porous and less compact flocs. Despite the high density of pyrite, its presence reduced sedimentation rates and overall thickening efficiency, especially at high flocculant doses, where saturation of active sites was evident. The results highlight that process efficiency depends not only on particle size or density, but also on the internal structure of the flocs and the specific interaction between the polymer and mineral surfaces, which has key implications for optimizing water recovery in tailings with high sulfide content.

Since these systems are inherently complex, computational tools have been essential to understand the molecular interactions between organic polymers and mineral surfaces [18,19,20,21]. In general, these studies have focused on aluminosilicate or silica surfaces, which, when present in aqueous suspensions, tend to hydrate and form hydroxyl groups that can be protonated or deprotonated depending on the pH of the medium [22,23]. On the other hand, sulfides such as pyrite are particularly sensitive to the species present in the system [24]; however, it is possible to perform simulations focused on non-oxidized or freshly exposed pyrite surfaces [24,25]. It is worth noting that, to date, the study of the interactions between pyrites and polymers using molecular dynamics has not been addressed in the literature.

This study evaluates the flocculation efficiency of synthetic tailings composed of quartz, kaolin, and varying proportions of pyrite using a high-molecular-weight anionic polyacrylamide in the presence of seawater at pH 8. The impact of mineralogical composition on the size, internal structure, and density of the formed flocs will be analyzed using tools such as FBRM, fractal dimension estimation, and molecular dynamics. It is important to note that under the conditions of this study (pH 8 in seawater), calcium and magnesium remain soluble and no precipitation of these ions is expected. This approach allows isolating the effect of pyrite on flocculant behavior under realistic salinity conditions. The results will allow more precise criteria to be established for the design and dosage of anionic polyacrylamide flocculants in seawater, contributing to the development of more efficient technologies for tailings management and water recovery in mining operations.

## 2. Materials and Methods

### 2.1. Materials

For the experiments, kaolin (Sigma-Aldrich, St. Louis, MO, USA), quartz (Donde Capo, Santiago, Chile), and pyrite (Ward’s Science, Rochester, NY, USA) particles were used. The density of pyrite was 5010 kg/m^3^, while the densities of kaolin and quartz were 2600 kg/m^3^.

The chemical composition of the seawater used to prepare the kaolin-quartz-pyrite (KQP) suspensions is presented in Table 1.

### 2.2. Sample Preparation

The flocculant used in this study was a partially hydrolyzed polyacrylamide (PAM) supplied by SNF (commercial name: SNF 704). This polymer presents an anionic characteristic with an average molecular weight of approximately 12–15 × 10^6^ g/mol and degree of hydrolysis of about 30%. The hydrolysis of amide groups into carboxylate groups provides active sites that enhance particle-polymer interactions through electrostatic attraction and bridging mechanisms. For experimental use, a stock solution of the polymer was prepared at a concentration of 1 g/L and kept under constant stirring for 24 h to ensure complete hydration and dissolution. The volume of this stock solution was subsequently diluted to reach a working concentration of 0.1 g/L, which was used in all flocculation tests. The flocculant dosage is reported in terms of grams of polymer per ton of dry solids (g/t), facilitating comparison with operating conditions used on an industrial scale.

**Figure 1:**
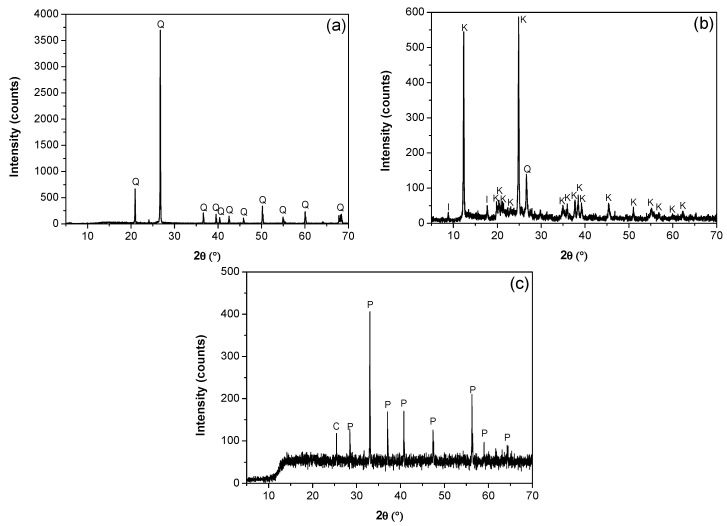
X-ray diffractograms. (**a**) Quartz (Q), (**b**) kaolin (I: illite, K: kaolinite, Q: Quartz), and (**c**) pyrite (C: chlorite, P: pyrite).

**Figure 2:**
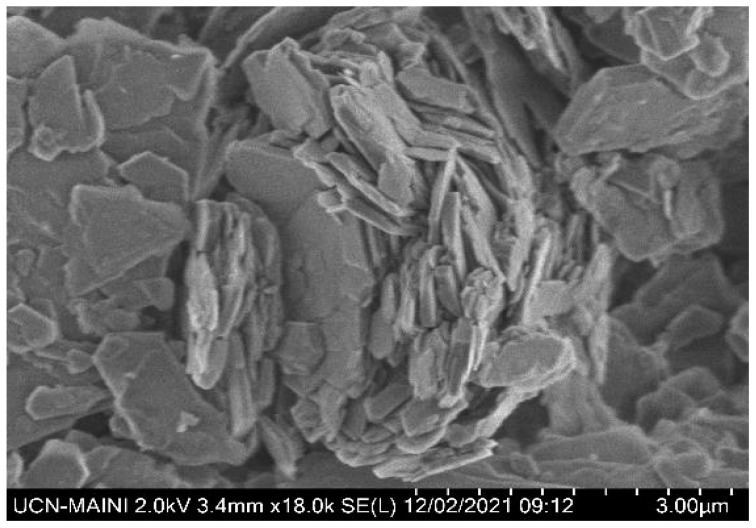
Morphology of kaolin particles obtained through SEM.

The mineralogical composition and purity of the kaolin, quartz, and pyrite minerals were determined using a D8 advance X-ray diffraction analyzer (Bruker AXS GmmbH, Karlsruhe, Germany) and TOPAS 3.0 software. Quartz was 99% pure by weight (Figure 1a). The diffraction analysis of the kaolin particles (Figure 1b) confirmed the presence of kaolinite, illite, and quartz, with kaolin being the majority phase, with a minor fraction of illite and quartz. The SEM micrograph in Figure 2 revealed a submicron population of kaolinite particles (<1 μm) with a hexagonal lamellar crystal structure undetectable with the focused beam reflectance measurement (FBRM) probe. A high-resolution SEM (Hitachi SU5000, ZRO Schottky emitter, Tokyo, Japan) equipped with Xflash 6I30 detectors (Bruker AXS GmbH, Karlsruhe, Germany) and a STEM detector (DEBEN UD Ltd., Suffolk, UK) was employed for this analysis. The pyrite was ground in a mortar and subsequently pulverized and sieved to obtain a particle size distribution between −75 µm and +38 µm. XRD analysis showed a majority composition of pyrite and a minority of chlorite (Figure 1c).

**Figure 3:**
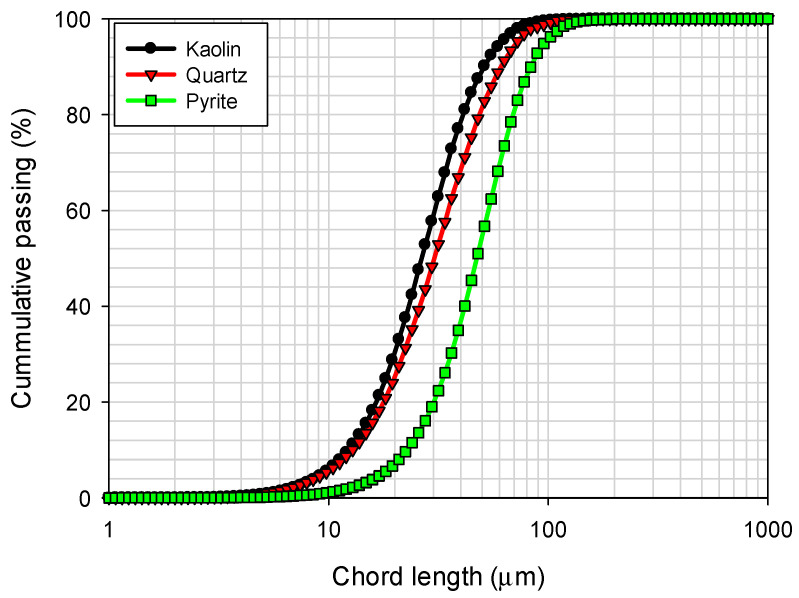
Particle size distribution of kaolin, quartz, and pyrite in seawater.

The chord length distributions for kaolin, pyrite, and quartz particles were obtained using focused beam reflectance measurement (FBRM) with a Particle Track E25 probe (Mettler Toledo, Columbus, OH, USA) and are presented in Figure 3. These measurements were performed in seawater. It is observed that kaolin and quartz particles show a significantly narrower size distribution compared to pyrite particles, which exhibit a broader distribution. In terms of percentiles, the analysis revealed that 10% of the particles had sizes less than d10 = 11.7 µm for kaolin, 12.8 µm for quartz, and 22.3 µm for pyrite, while 90% of the particles had d90 = 50.7 µm for kaolin, 60.9 µm for quartz, and 82.3 µm for pyrite.

### 2.3. Methods

#### 2.3.1. Sedimentation-Flocculation Tests

Synthetic kaolin-quartz-pyrite tailings suspensions were prepared at a solids concentration of 10% by weight using seawater as the medium. The proportion of kaolin was kept constant at 10%, while the pyrite concentration was adjusted between 0 and 10%, with quartz being added to the mixture. The suspensions were vigorously stirred for 10 min at 500 rpm using a 30 mm diameter polyethylene tetrafluoride (PTFE) turbine stirrer, axially positioned in a 100 mm diameter flocculation vessel with a 1 L capacity. The stirrer was positioned 20 ± 1 mm above the bottom of the vessel. The stirring speed was subsequently reduced to 200 rpm, and SNF 704 flocculant was added at doses of 10 and 30 g/t. The prepared suspensions were used in batch sedimentation tests and floc characterization.

In batch sedimentation tests, after 30 s of mixing between the suspension and the flocculant, the suspensions were poured into removable cylindrical vessels with a 300 cm^3^ capacity and 35 mm internal diameter. The vessels were turned five times to homogenize the mixture before video recording, which allowed the sedimentation rate to be measured.

For floc characterization, an FBRM (focused beam reflectance measurement) probe was used. The probe was inserted vertically into the suspension, 10 mm above the PTFE stirrer and 20 mm from the central axis. A Particle Track G400 with FBRM technology (Mettler Toledo, Columbus, OH, USA) was used. The probe generated a focused laser that scanned a circular path at a tangential velocity of 2 m/s through a sapphire window. Upon intercepting a particle, the laser emitted a backscatter signal proportional to the particle’s chord length, enabling real-time monitoring of changes in size and number of fine and coarse particles in suspension. These data facilitated the evaluation of floc formation and evolution under different experimental conditions.

#### 2.3.2. Fractal Dimension

Fractal dimension (Df) is an essential tool for describing the structure and complexity of the flocs formed during the thickening process. This value ranges from 1 to 3, where 1 indicates a simple one-dimensional structure (such as a line) and 3 represents a completely solid three-dimensional shape (such as a sphere). Fractal dimension helps characterize objects that do not have a simple, regular shape, such as flocs formed during sedimentation processes, which often exhibit irregular and complex structures.

Flocs with high fractal dimension have a more compact, dense structure and are resistant to fragmentation under shear conditions. These flocs tend to have greater stability, which improves their sedimentation and consolidation capacity. Conversely, flocs with a low fractal dimension are usually more open, porous, and have a low density, making them more susceptible to fragmentation. These flocs with lower fractal dimensions have a more dispersed structure, which can make them challenging to compact in thickeners.

Fractal dimension also influences the efficiency of the flocculation process. Flocs with high fractal dimensions are generally easier to treat and more effective at retaining fine particles, improving water recovery. This is because their greater compaction favors rapid sedimentation and the formation of a denser layer at the base of the thickener.

The fractal dimension of the flocs is determined using the equation proposed by Heath et al. [27].

(1) $$U_{h}=\frac{d_{agg}^{2}g(\rho_{s}{-\rho }_{l}){\left(\frac{d_{agg}}{d_{p}}\right)}^{D_{f}-3}}{18µ}{\left(1-\phi_{s}{\left(\frac{d_{agg}}{d_{p}}\right)}^{3-D_{f}}\right)}^{4.65}$$

where $U_{h}$ is the hindered settling velocity in m/s, $d_{p}$ and $d_{agg}$ are, respectively, the average particle size and the average floc size after a flocculation time, $\rho_{s}$ and $\rho_{l}$ are, respectively, the densities of the solid and liquid phases, $\phi_{s}$ is the volume fraction of the solid, µ is the viscosity of the fluid and $D_{f}$ is the fractal dimension of mass. $D_{f}$ was determined using the hindered settling velocity from Section 2.3.1, and the squared weighted mean chord length obtained with the FBRM probe was used for the average floc size. The values are presented in Table 2.

The apparent density of the flocculated flocs was calculated using an equation proposed by Kranenburg [28], which establishes a relationship between the densities of the solid ($\rho_{s}$), liquid ($\rho_{l})$ and floc ($\rho_{agg})$, as well as the diameters of the primary particles ($d_{p}$) and flocs ($d_{agg}$), and the previously determined fractal dimension ($D_{f}$). This formula, based on principles of fractal geometry, facilitates a more accurate estimation of the floc behavior during the sedimentation process. By considering both structural and compositional parameters, this approach offers a valuable tool for understanding the dynamics of floc settling in complex syste.

(2) $$\left(\rho_{agg}-\rho_{l}\right)=\left(\rho_{s}-\rho_{l}\right){\left(\frac{d_{agg}}{d_{p}}\right)}^{Df-3}$$

It should be noted that in this study a single average particle diameter ($d_{p}$) was used instead of the complete primary particle size distribution, which means that the obtained fractal dimension values should be interpreted qualitatively rather than as absolute metrics. For transparency, the average particle diameters employed in each calculation are provided in Table A1 in the Appendix A. These values were similar across the different experimental conditions, supporting the consistency of the comparative analysis performed in this work.

#### 2.3.3. Zeta Potential

Suspensions composed of quartz, kaolin, and pyrite were prepared with a solids concentration of 1% by weight using seawater as the medium. These suspensions were evaluated in the absence and presence of SNF 704 flocculant (1 ppm). Lime was used to adjust the pH to 8, and homogeneous conditions were ensured in the samples before proceeding with the measurements. The zeta potential of the particles was obtained using a Litesizer 500 (Anton Paar, Graz, Austria), which employs the principle of electrophoretic light scattering using the CmPALS (Continuously Monitored Phase Analysis Light Scattering) technique. The measurements were carried out in an Omega Zeta cell (Anton Paar, Graz, Autria) controlled by Kalliope softwate, version 3.8.2 (Anton Paar, Graz, Austria), specifically designed to provide precision in colloidal systems. The experimental conditions were controlled at a temperature of 20 °C and a voltage of 220 V. This data allowed us to study the effect of the flocculant and the surface chemical interactions of the particles in an alkaline environment, like the conditions present in mining flocculation processes.

#### 2.3.4. Molecular Dynamics Simulations

Molecular dynamics (MD) simulations were carried out to complement experimental studies and provide a molecular-level insight into the interactions between polymers and mineral surfaces. Calculations were performed using GROMACS version 2022.1 software running on a high-performance cluster with GPU cards. The modeled system included a crystalline surface (Quartz, Kaolinite, and Pyrite), 1 molecule of anionic polyacrylamide (48 monomers with 25% anionicity), in a synthetic seawater solution (NaCl 28 g/L, MgCl_2_ 4.8 g/L, CaCl_2_ 1.1 g/L). Interactions were described using the CLAYFF force field [29] for the kaolinite and quartz surfaces, UFF [24] for pyrite, GAFF [30] for the polymer, Li/Merz [31,32] for the ions, and SPCE [33] for water. The initial configuration was built using proprietary codes, ensuring a realistic packing of the system without non-physical overlaps. Subsequently, a system preparation process was applied, and adsorption configurations of the polymer were generated from one of its ends (END) or in the middle of its chain (MID). For more details on this methodology, see [34]. The adsorption analysis time was 10 ns with these configurations, and 3 repetitions were performed per system and configuration studied. LINCS [35], PME [36], Nose-Hoover [37,38], and Parrinello-Rahman [39] algorithms were used to ensure a rigorous simulation. The subsequent analysis included the calculation of density profiles, the number of contacts between the polymer and surface, and hydrogen bonds. For this purpose, tools integrated into GROMACS (2022.1) and custom scripts in Python (3.6.8) and MATLAB (R2024b) were used.

## 3. Results

### 3.1. Sedimentation Tests

In mining, sedimentation efficiency is crucial to optimize solids concentration in the thickener, which in turn improves water utilization in operations. Factors such as the initial sedimentation rate, pH, water quality, and solids proportion and concentration are key determinants of thickening process performance, directly influencing operating costs.

**Figure 4:**
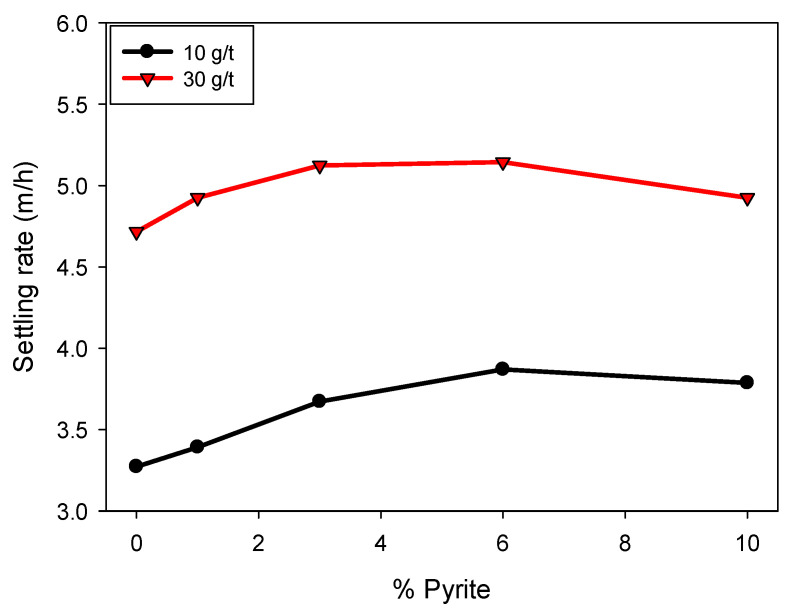
Effect of pyrite on the sedimentation rate in kaolin-quartz-pyrite tailings in seawater at pH 8, with flocculant dosages of 10 and 30 g/t. Mixing intensity, 200 rpm; solids concentration by weight, 10%.

Figure 4 shows that the sedimentation rate increases with increasing flocculant dosage. This is attributed to the formation of cationic bridges between the particles and the polymer, favoring the generation of larger, more massive flocs, which accelerates sedimentation. Likewise, an increasing trend in sedimentation rate is observed with increasing pyrite proportion, up to a content of 6%, at which point the effect stabilizes. For example, at a dosage of 30 g/t, the sedimentation velocity increased from 4.7 to 5.1 m/h between 0 and 6% pyrite, remaining constant at higher proportions. Similar behavior was observed at a dosage of 10 g/t, where the sedimentation velocity varied from 3.2 to 3.8 m/h in the same range in pyrite proportion, with no significant changes at 10%. This non-monotonic behavior can be explained by two combined effects: (i) the higher density of pyrite particles, which initially enhances settling when incorporated into flocs, and (ii) the high affinity of pyrite for the flocculant, which at higher proportions leads to a competitive effect where most of the polymer adsorbs onto pyrite surfaces, reducing its availability to interact with clay particles. This competition results in a slight detriment in sedimentation rate at elevated pyrite contents, reflecting the limited efficiency of floc formation under these conditions.

### 3.2. Flocculation Kinetics as a Function of Pyrite Content

**Figure 5:**
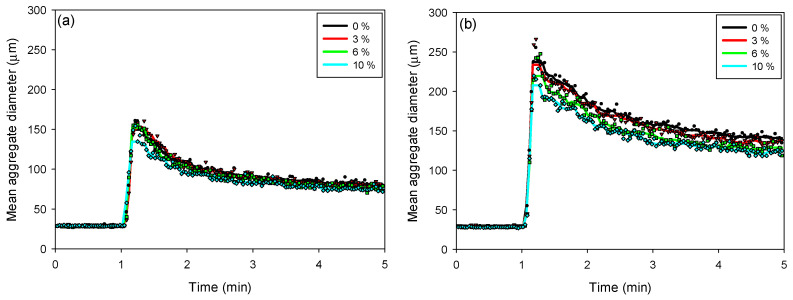
Flocculation kinetics as a function of the pyrite percentage in kaolin-quartz-pyrite suspensions, considering flocculant doses of 10 g/t (**a**) and 30 g/t (**b**). Mixing intensity: 200 rpm, solids concentration by weight: 10%, pH: 8. (The symbols ● 0%, ▲ 3%, ■ 6% and ◆ 10% denote experimental data).

The kinetics of floc aggregation, fragmentation, and restructuring in flocculated kaolin-quartz-pyrite suspensions were analyzed using kinetic profiles obtained with the FBRM probe. The SNF 704 flocculant dosages used were 10 g/t (Figure 5a) and 30 g/t (Figure 5b). In both cases, the addition of the flocculant caused a rapid increase in floc size, reaching a maximum value a few seconds after the onset of flocculation. Both the flocculant dosage and the proportion of pyrite in suspension significantly influenced the maximum floc size.

For both flocculant dosages, the maximum floc size decreased with increasing pyrite proportion. At a dosage of 10 g/t, the maximum size varied from 162 to 135 µm as the pyrite proportion increased from 0 to 10%. With an increase in flocculant dosage to 30 g/t, the maximum floc size decreased from 256 to 208 µm, for pyrite proportions between 0 and 10%, respectively. At higher pyrite proportions, the flocs tend to be smaller, as pyrite can modify surface interactions and reduce floc stability, favoring their fragmentation and compaction.

As the pyrite proportion increases (from 0 to 10%), the flocs tend to become smaller and more compact, facilitating sedimentation. This suggests that floc growth is less efficient, as pyrite, due to its high density and surface properties, interferes with the formation of large flocs. For proportions greater than 6% pyrite, it is observed that the sedimentation rate stabilizes, indicating that the pyrite is in the saturation zone, which plays a determining role in the sedimentation performance, since its presence not only affects the size and structure of the flocs, but also the ability to settle efficiently.

### 3.3. Chord Length Distributions (CLD)

**Figure 6:**
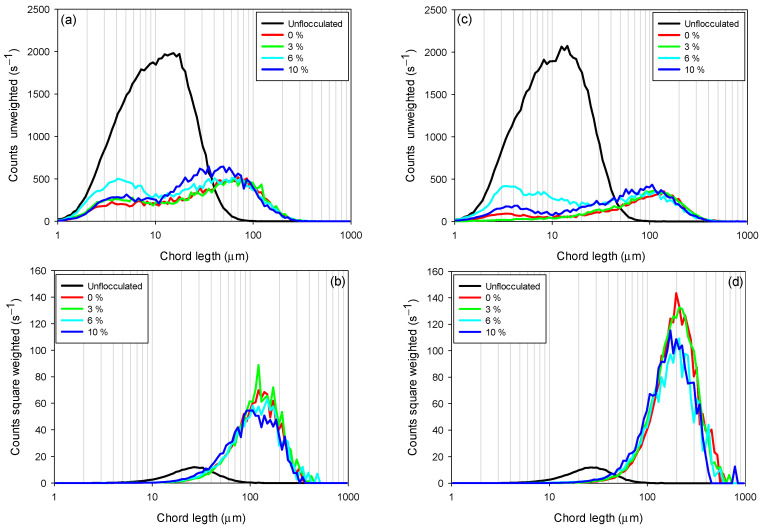
Variation in chord length distributions (CLDs) as a function of pyrite content in seawater at pH 8. (**a**) Unweighted CLD for KQP under a dosage of 10 g/t, (**b**) square-weighted CLD for KQP under a dosage of 10 g/t, (**c**) unweighted CLD for KQP at a dosage of 30 g/t, and (**d**) square-weighted CLD for KQP at a dosage of 30 g/t. Stirring speed of the mixture, 200 rpm; solids concentration by weight, 10%; pH 8.

The chord length obtained with the FBRM probe is a key measure for characterizing the size of flocs or particles in suspensions, regardless of whether they are flocculated or not, due to its ability to provide an accurate measurement of the maximum particle extension, especially in systems with irregular, elongated, or complex shapes such as clays. This is especially relevant in flocculated systems, where flocs do not have a uniform geometry and their sedimentation and rheological behavior are strongly influenced by their shape. Figure 6 shows the chord length distributions in unweighted (Figure 6a,c) and quadratically weighted (Figure 6b,d) modes, for tailings composed of kaolin-quartz-pyrite in seawater, considering different pyrite proportions (0–10%). Unweighted CLD provides detailed information on smaller flocs, while quadratically weighted CLD includes information related to larger flocs, where it is more sensitive to changes in the coarse fraction [40,41].

Figure 6a,c show that increasing the proportion of pyrite in the suspension (from 0 to 10%) increases the amount of unflocculated fine particles (2–10 µm). This phenomenon indicates that the presence of pyrite does not favor the flocculation of fine particles, negatively impacting process efficiency for both flocculant dosages (10 and 30 g/t). For example, at a dosage of 10 g/t, the suspensions with 0 and 3% pyrite had the lowest fine particle counts, reaching maximum peaks of 208 and 260 s^−1^, respectively. In contrast, for pyrite suspensions of 6 and 10%, peak particle counts were recorded at 500 and 288 s^−1^, respectively. Similarly, for a dosage of 30 g/t, the peak particle counts for 0 and 3% pyrite suspensions were 90 and 15 s^−1^, respectively, while for suspensions with 6 and 10% proportions, they were 421 and 185 s^−1^, respectively. The particularly high fine particle count at 6% pyrite, observed at both dosages, suggests a critical competition effect: due to the strong affinity of pyrite for the flocculant, a significant fraction of polymer chains adsorbs preferentially on pyrite surfaces at this intermediate proportion. As a result, fewer chains remain available to bridge clay particles, leading to inefficient capture of fines and the non-monotonic distribution observed.

Flocculant dosage also significantly affects fine particle capture. For all pyrite proportions studied, fine particle counts decreased with increasing flocculant dosage. For example, while maintaining a constant 6% pyrite proportion, a flocculant dosage of 10 g/t showed a higher fine particle count (500 s^−1^) compared to a dosage of 30 g/t, which showed a count of 421 s^−1^. This indicates an improvement in flocculation efficiency with increasing flocculant dosage.

The square-weighted chord length distributions (Figure 6b,d) showed that the size of coarse flocs increased with increasing flocculant dosage. However, this size decreased with increasing percentage of pyrite (from 0 to 10%). This behavior is presented in the figures as a shift in the curves from left to right and a decrease in peak counts for pyrite proportions from 10 to 0%. This indicates a reduction in floc size, primarily at high pyrite proportions, where the strong affinity of pyrite for the flocculant promotes the formation of polymer trains on its surface. This preferential adsorption limits floc growth by reducing the availability of active sites for interaction with clay particles, thereby preventing effective particle.

This confirms that high pyrite proportions impair the initial capture of fine particles. Therefore, the flocs formed are of low density and more fragile, justifying the stabilization of sedimentation velocities at high pyrite proportions (10%).

### 3.4. Fractal Dimension and Density of Flocs

**Figure 7:**
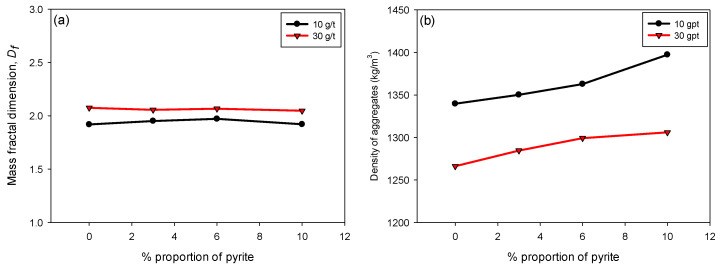
(**a**) Fractal dimension Df and (**b**) floc density, varying the pyrite content and considering flocculant doses of 10 and 30 g/t of SNF 704, with 30 s of flocculation, percentage of solids by weight of 10%, and pH 8.

Figure 7a illustrates the variation in Df as a function of the pyrite percentage (0–10%) of flocculated KQP suspensions in seawater for flocculant doses of 10 and 30 g/t. It is observed that Df remains constant throughout the pyrite percentage range. This behavior indicates that, despite changes in the pyrite proportion, there is no significant effect on the fractal structure of the flocs. Pyrite may not significantly alter the morphology of the flocs in terms of their structural complexity, which is reflected in the stability of Df.

Figure 7b shows the variation in the density of the flocs formed as a function of the pyrite content in suspension. Unlike what was observed for Df, the density of the flocs increased linearly with the pyrite proportion for both flocculant doses. This increase can be attributed to the higher specific gravity of pyrite compared to kaolin and quartz minerals, which contributes to a greater densification of the flocs by increasing their volumetric fraction. Additionally, it is observed that at a dose of 30 g/t, the flocs presented lower densities compared to those formed at 10 g/t. This behavior is due to a greater inclusion of interstitial water trapped within the larger and less compact flocs [42,43].

Although floc density is typically expected to increase with higher fractal dimension, our results show that at higher polymer dosages (30 g/t), flocs exhibited greater Df but lower density. This behavior can be explained by the simultaneous capture of ultrafine particles and the formation of extended polymer–particle networks. These structures appear more space-filling, which increases Df, but they also generate internal heterogeneities and porosity that entrap water. Consequently, the flocs display higher fractal dimensions while their overall floc density decreases.

### 3.5. Effect of Flocculant on Particle Zeta Potential

Table 3 presents the zeta potentials of kaolin, quartz, and pyrite surfaces in the presence and absence of SNF 704 flocculant at pH 8 in seawater. In the absence of flocculant, kaolin and quartz exhibited zeta potential values of −13.7 and −7.4 mV, respectively, while pyrite had a positive zeta potential of approximately 5.3 mV. In the presence of flocculant, kaolin and pyrite exhibited different behaviors, primarily reflecting the combined effects of particle surface charge, cation adsorption, and interaction with the anionic polyacrylamide SNF 704.

- Pyrite: The presence of the anionic flocculant significantly decreased the zeta potential of pyrite, from a positive value of 5.3 mV to −4.5 mV. In the absence of flocculant, the positive surface charge can be explained by the preferential adsorption of monovalent and divalent cations, such as Na^+^, K^+^, Ca^2+^, and Mg^2+^ [44], abundant in seawater. These ions can form complexes that adsorb on the mineral surface. With the addition of the flocculant, the mineral charge is reversed. This change is attributed to the adsorption of the polymer, which induces a neutralization of the positive charges on the mineral surface, decreasing electrostatic repulsions and promoting aggregation.
- Kaolin: In the absence of flocculant, the zeta potential of kaolin was negative at −13.7 mV, a value consistent with its surface anionic nature under pH ≈ 8 conditions in seawater [45]. This negative charge is mainly caused by the deprotonation of hydroxyl groups on the edges and faces of mineral particles, as well as isomorphic substitution within the crystal structure. These factors generate a negatively charged surface. After the addition of anionic flocculant, the zeta potential decreased to −20.3 mV. This indicates that the anionic polymer interacts with the kaolin surface by adsorbing its chains, modifying the structure of the electrical double layer. As a result, the density of negative charges exposed to the continuous phase increases, which translates into a greater negativity of the zeta potential.
- Quartz: The negative zeta potential of quartz at pH 8 was −7.4, a value consistent with its negative surface charge generated by the deprotonation of silanol groups. Upon incorporation of the flocculant, the zeta potential decreased slightly to −8.7 mV, suggesting low adsorption of the polymer by quartz, probably limited by its low surface charge density, which limits the interaction between the polymer and the mineral surface.

### 3.6. Molecular Dynamics Adsorption Study

Molecular dynamics simulations of a polyacrylamide (PAM) chain were performed in seawater at pH 8 in the presence of three surfaces: pyrite, kaolinite, and quartz. Two initial polymer configurations were evaluated: END (adsorption at the end) and MID (adsorption at the center of the chain).

**Figure 8:**
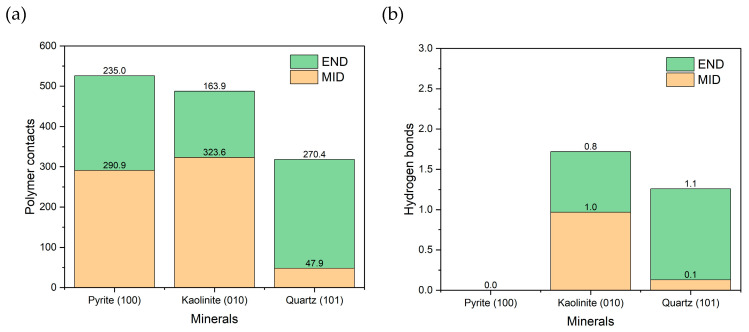
(**a**) Number of polymer-surface contacts at a cut-off radius of 0.5 nm. (**b**) Hydrogen bonds between the polymer and surface. Results are in seawater at pH 8.

As shown in Figure 8a, pyrite had the most significant number of contacts with the PAM, closely followed by kaolinite, and to a lesser extent, quartz. The results follow the trends of previous work [46], where kaolinite had the greatest adsorption, followed by quartz. The pyrite result is interesting because only one unoxidized surface was present, yet stable adsorption was still generated. When analyzed by configuration, quartz showed greater affinity for END, while kaolinite favored the MID configuration. In the case of pyrite, no clear preference was observed between END and MID. Figure 8b shows the number of hydrogen bonds formed. No such interactions were evident with pyrite, suggesting that specific bonds do not mediate their adsorption. In contrast, kaolinite and quartz formed hydrogen bonds in proportions consistent with the observed contacts, confirming their role in the interaction with the polymer.

**Figure 9:**
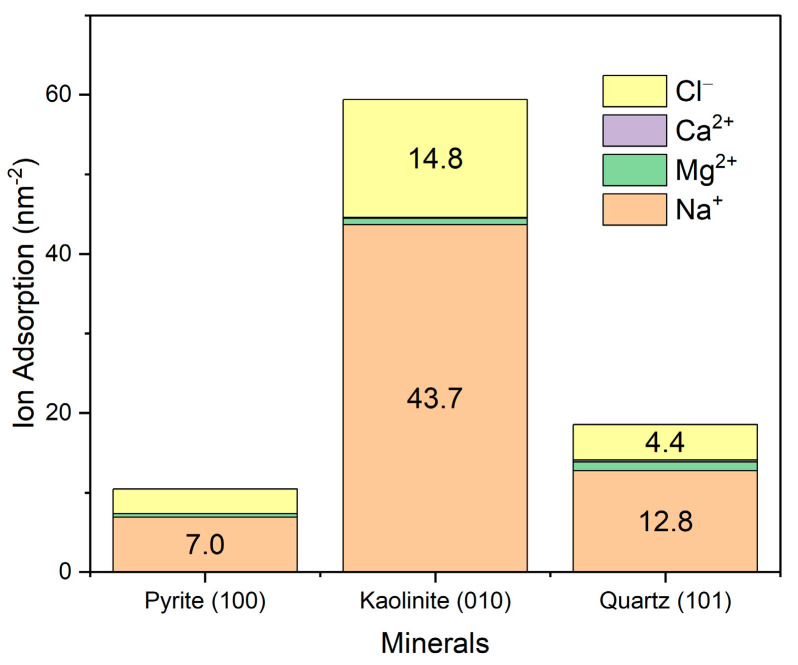
Surface adsorption of ions from seawater onto mineral surfaces. Results are in seawater at pH 8.

The adsorption of primary ions in seawater was evaluated by integrating the density profile, assuming the system had achieved a stable ionic distribution. As shown in Figure 9, ions exhibit the highest adsorption on the (010) edge surface of kaolinite, followed by quartz and, to a lesser extent, pyrite. It is important to note that these results correspond to specific surface planes, which can significantly influence the observed adsorption. In the case of quartz and pyrite, surfaces with close to isotropic symmetry were studied, while kaolinite displays a strongly anisotropic character. In particular, the (010) surface of kaolinite is recognized for its high reactivity and charge density, in contrast to its basal (001) planes, which show a much lower ionic adsorption capacity. Therefore, the overall adsorption of ions on kaolinite could be overestimated if only its exposed edges are considered. Regarding the adsorbed ionic composition, sodium was the most retained ion, followed by magnesium, while calcium and chloride showed significantly lower adsorptions.

**Figure 10:**
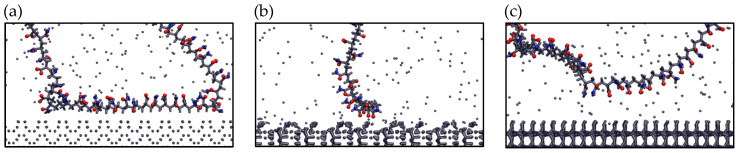
Configurations of anionic PAM adsorbed on (**a**) Pyrite (100), (**b**) Kaolinite (010), and (**c**) Quartz (101) surfaces—results in seawater at pH 8. Blue spheres are Nitrogen atoms and red spheres are oxygen atoms.

Figure 10 presents the final adsorption configurations of the anionic polymer PAM on the three mineral surfaces. These visualizations confirm the trends observed in previous analyses. On pyrite, adsorption occurs primarily through the aliphatic segments of the polymer, suggesting a more versatile behavior less dependent on charged functional groups. This type of interaction could be favored by the less polar nature of the surface. In contrast, on kaolinite and quartz, adsorption occurs mainly through carboxylic and amide groups of PAM, in line with their higher surface charge density (in the case of kaolinite) and presence of hydroxyl groups. These results show that polymer adsorption depends not only on surface charge but also on the structure and local chemistry of the surface, making it difficult to predict from simple parameters.

## 4. Conclusions

In this study, the impact of pyrite on the flocculation and sedimentation efficiency of clayey tailings in seawater at pH 8 was evaluated. The results provided significant information for the optimization of thickening processes containing this mineral.

- The mineralogical composition of pyrite influences flocculation efficiency in seawater, directly modifying the aggregation kinetics, floc size, and sedimentation efficiency due to its surface properties and high density. Increasing the proportion of pyrite reduced the maximum floc size and increased the amount of unflocculated fine particles for both flocculant doses (10 and 30 g/t).
- The fractal dimension of the flocs remained constant with increasing pyrite, suggesting that the internal structural complexity of the flocs was not significantly altered. However, the density of the flocs increased linearly with the proportion of pyrite, which explains the higher intrinsic density of this mineral.
- Pyrite adsorbs the polymer through uncharged sections of the chain, making it less sensitive to pH or salinity conditions than other minerals such as clays or oxides. It can also cause train-type adsorption, where the polymer flattens on the mineral surface, preventing the flocculant from interacting with kaolin particles and affecting the sedimentation process, especially for pyrite proportions greater than 6%.
- Simulations showed that pyrite has a high affinity for PAM polymer, with adsorption even in aliphatic regions. This behavior is consistent with experimental results, where the presence of pyrite favors the formation of larger flocs, attributed to its ability to retain the polymer and facilitate the union between particles.

## Figures and Tables

**Table 1:** Typical chemical composition of seawater [26].

Salt	Concentration (g/L)
NaCl	24.53
MgCl_2_·6H_2_O	11.10
Na_2_SO_4_	4.09
CaCl_2_	1.16
KCl	0.69
NaHCO_3_	0.20
KBr	0.10
H_3_BO_3_	0.03

**Table 2:** Parameters used for all slurries.

Parameter	Values
$d_{p}\;(\mu m)$	28.6 ± 0.6
$\rho_{solid}\;(Kgm^{-3})$	2692.5 ± 51.5
$\rho_{liquid}\;(Kgm^{-3})$	1025
$g\;(ms^{-2})$	9.81
$\mu_{liquid}\;(Nsm^{-2})$	0.001021
$\phi_{solid}$	0.040

**Table 3:** Zeta potential of kaolin, quartz, and pyrite surfaces in the absence and presence of flocculant in seawater at pH 8.

	Unflocculated	Flocculated
Kaolin	−13.7	−20.3
Pyrite	5.3	−4.5
Quartz	−7.4	−8.7

## Data Availability

The original contributions presented in the study are included in the article; further inquiries can be directed to the corresponding author.

## Appendix A

The appendix provides the additional parameters used for the calculation of fractal dimension and floc density.

**Table A1:** Additional parameters used for the calculation of fractal dimension and floc density, considering a flocculation time of 30 s and a mixing speed of 200 rpm at pH 8.

Proportion of Pyrite (%)	Flocculant Dosage (g/t)	$d_{p}$ (µm)	$\rho_{s}$ (kg/m^3^)	$\rho_{l}$ (kg/m^3^)	$\phi_{s}$
0	10	29.2	2630	1025	0.042
3	10	28.9	2670	1025	0.041
6	10	28.1	2710	1025	0.040
10	10	29.0	2760	1025	0.040
0	30	28.4	2630	1025	0.042
3	30	29.6	2670	1025	0.041
6	30	28.2	2710	1025	0.040
10	30	27.9	2760	1025	0.040
